# Identification of TRPM2 as a Marker Associated With Prognosis and Immune Infiltration in Kidney Renal Clear Cell Carcinoma

**DOI:** 10.3389/fmolb.2021.774905

**Published:** 2022-01-05

**Authors:** Lei Sun, Zijun Zhang, Hang Zhao, Miaoyun Qiu, Ying Wen, Xiaoqiang Yao, Wai Ho Tang

**Affiliations:** ^1^ Institute of Pediatrics, Guangzhou Women and Children’s Medical Centre, Guangzhou Medical University, Guangzhou, China; ^2^ School of Biomedical Sciences, The Chinese University of Hong Kong, Hong Kong SAR, China; ^3^ Li Ka Shing Institute of Health Science, The Chinese University of Hong Kong, Hong Kong SAR, China

**Keywords:** TRPM2, KIRC, immune infiltration, prognosis, T cell activation

## Abstract

TRPM2 (transient receptor potential melastatin-2), a Ca^2+^ permeable, non-selective cation channel, is highly expressed in cancers and regulates tumor cell migration, invasion, and proliferation. However, no study has yet demonstrated the association of TRPM2 with the prognosis of cancer patients or tumor immune infiltration, and the possibility and the clinical basis of TRPM2 as a prognostic marker in cancers are yet unknown. In the current study, we first explored the correlation between the mRNA level of *TRPM2* and the prognosis of patients with different cancers across public databases. Subsequently, the Tumor Immune Estimation Resource (TIMER) platform and the TISIDB website were used to assess the correlation between TRPM2 and tumor immune cell infiltration level. We found that 1) the level of TRPM2 was significantly elevated in most tumor tissues relative to normal tissues; 2) TRPM2 upregulation was significantly associated with adverse clinical characteristics and poor survival of kidney renal clear cell carcinoma (KIRC) patients; 3) the level of TRPM2 was positively related to immune cell infiltration. Moreover, TRPM2 was closely correlated to the gene markers of diverse immune cells; 4) a high TRPM2 expression predicted worse prognosis in KIRC based on different enriched immune cell cohorts; and 5) TRPM2 was mainly implemented in the T-cell activation process indicated by Gene Ontology (GO) function and Kyoto Encyclopedia of Genes and Genomes (KEGG) pathway enrichment analysis. In conclusion, TRPM2 can serve as a marker to predict the prognosis and immune infiltration in KIRC through the regulation of T-cell activation. The current data may provide additional information for further studies surrounding the function of TRPM2 in KIRC.

## Introduction

Transient receptor potential (TRP) ion channels are a family of membrane proteins that play diverse physiological and pathological roles. Previous studies have indicated that TRP channels have a strong diagnostic potential for various cancer types, especially in head and neck, kidney, and lung cancers, in which clinically useful diagnostic markers are not available (Park et al., 2016). Aberrant regulation of TRP channels results in various diseases, including numerous types of cancer.

TRPM2 is a Ca^2+^-permeable, non-selective cation channel activated by ADP-ribose (ADPR), temperature, oxidative stress, and Ca^2+^ (Sumoza-Toledo and Penner, 2011b). The activation of TRPM2 results in the transport of Ca^2+^ across the plasma membrane and the release of cytosolic Ca^2+^ from organelles of the endoplasmic reticulum store and lysosome (Lange et al., 2009; Sun et al., 2012; Li F. et al., 2016). TRPM2 is ubiquitously distributed in the body, especially highly expressed in many energy-demanding tissues, including the brain, heart, and vasculature. The physiological functions of TRPM2 include insulin secretion in the pancreas, warm sensitivity in neurons (Tan and McNaughton, 2016; Paricio-Montesinos et al., 2020), and the induction of dendritic cell maturation and chemotaxis (Sumoza-Toledo et al., 2011a). TRPM2 expressed in immune cells, such as macrophages and polymorphonuclear neutrophils (PMNs), is crucial for regulating the bactericidal activity of phagocytic cells and PMN migration in tissues (Mittal et al., 2017).

Recent studies have demonstrated that TRPM2 was highly expressed in melanoma, breast cancer, prostate cancer, tongue cancer, neuroblastoma, and kidney cancer (Hopkins et al., 2015; Park et al., 2016). Knockdown/inhibition of TRPM2 impaired mitochondrial function and autophagy, reduced cellular bioenergetics, and increased the levels of reactive oxygen species (ROS), resulting in decreased tumor proliferation and/or viability in many malignancies, suggesting a role of TRPM2 in cancer cell propagation and growth (Hopkins et al., 2015; Miller, 2019). Next, we questioned whether TRPM2 could be used as a prognostic marker in cancers. We screened the mRNA level of *TRPM2* in the Gene Expression Profiling and Interactive Analysis (GEPIA) and TISIDB databases and found that the expression of TRPM2 was significantly enhanced in several cancers; of these, kidney renal clear cell carcinoma (KIRC) was the most TRPM2-relevant cancer with respect to tumor subtypes, cancer stages, nodal metastasis, and tumor grades.

KIRC is the most common histological subtype that accounts for almost 90% of all kidney tumors (Zeng et al., 2020; Arora and Limaiem, 2021). Currently, surgical resection is the first-line treatment for KIRC. However, local recurrence or distant metastasis persists in 25% of patients with “local” disease following curative nephrectomy (Wang et al., 2020). Therefore, KIRC is one of the most aggressive kidney cancers mainly due to the high risk of tumor recurrence, metastasis, chemotherapy, and radiotherapy resistance (Linehan, 2012). Although multidisciplinary synthetic therapy has been used to treat KIRC, the prognosis and clinical outcomes have been unsatisfactory. Moreover, there are no credible predictive markers for the prognosis and treatment of individual sensitivity or resistance in KIRC, although some prognostic factors associated with the survival of KIRC patients have been described (Park et al., 2016; Marquardt et al., 2021). To the best of our knowledge, there have been no studies in the literature that investigated the role of TRPM2 in kidney cancers. Only one article has mentioned that the overexpression of TRPM2 might be used as a diagnostic marker for kidney cancer in terms of sensitivity and specificity, indicating strong diagnostic potential of TRPM2 (Park et al., 2016). In addition, a high expression of TRPM2 was closely associated with poor prognosis in bladder, head and neck, liver, and lung cancers (adenocarcinoma) (OR = 14.260–389.563), implying that TRPM2 significantly affects cancer progression (Park et al., 2016).

Features of the tumor immune microenvironment (TIME) are highly heterogeneous and have emerged as critical biomarkers in predicting the efficacy of and the response to systemic therapy. KIRC is one of the most immune-infiltrated tumors in pan-cancer comparisons (Vuong et al., 2019). Recent studies have considered KIRC as an immunogenic tumor with immune dysfunction partly as a result of the infiltration of immune-inhibitory cells, such as regulatory T cells (Tregs) and myeloid-derived suppressor cells, into the tumor microenvironment (TME) (Diaz-Montero et al., 2020). The association of TRPM2 with immune landscapes in KIRC may have clinical implications in defining distinct classes and subclasses of TIME.

In the present study, we described the performance of TRPM2 as a predictive marker of KIRC on its association with immune infiltration and then further explored the implicated mechanism based on relevant clinical background. Firstly, we used the Tumor Immune Estimation Resource (TIMER) and Gene Expression Profiling and Interactive Analysis (GEPIA) databases to systematically analyze the mRNA level of *TRPM2* in different types of tumors and assessed its prognostic value using the Kaplan–Meier plotter and the GEPIA database. Furthermore, we used the TIMER and TISIDB databases to assess the correlation between TRPM2 and the abundance of tumor-infiltrating immune cells in the TME. Finally, TRPM2-correlated genes were enriched using Gene Ontology (GO) and Kyoto Encyclopedia of Genes and Genomes (KEGG), indicating that TRPM2 may be largely involved in T-cell activation in KIRC.

## Materials and Methods

### TIMER Database Analysis

TIMER (http://timer.comp-genomics.org/) is a comprehensive resource for systematic analysis of immune infiltrates across diverse cancer types. This platform provides four modules for investigating the association between the immune infiltrates and genetic or clinical features and four modules for exploring cancer-related associations in The Cancer Genome Atlas (TCGA) cohorts (Li B. et al., 2016; Li et al., 2017; Li et al., 2020).

We used the Gene_DE module to investigate the mRNA expression of *TRPM2* in different cancer types and matched normal tissues across all TCGA cancer types. The level of TRPM2 was expressed as log2 TPM, and the significance was determined by differential gene expression analysis using the edgeR software package.

We also used this platform to evaluate the correlation of the expression level of TRPM2 with tumor purity and tumor infiltration of B cells, CD4^+^ T cells, CD8^+^ T cells, neutrophils, macrophages, and dendritic cells. A heatmap of Spearman’s correlations between the level of TRPM2 and immune cell infiltration across diverse cancer types was also generated using the TIMER database. Furthermore, the correlation between TRPM2 expression and the gene markers of immune cells identified the potential subtypes of infiltrating immune cells.

### GEPIA

**FIGURE 1:**
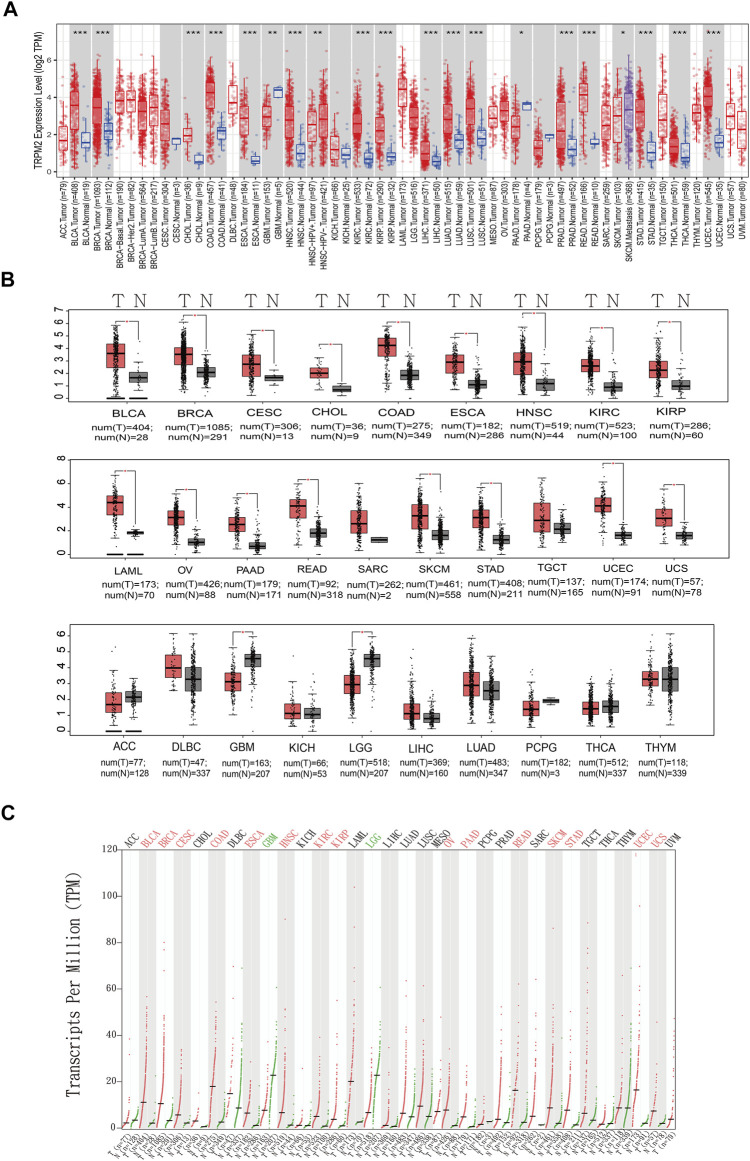
Expression of TRPM2 in different cancers. **(A)** Box plot showing the expression level of TRPM2 in different cancer types compared to adjacent normal tissues determined based on data from The Cancer Genome Atlas (TCGA) using TIMER 2.0. The significance computed by Wilcoxon’s test was annotated by the *number of stars*. **(B)** Box plots displaying the expression of TRPM2 in different cancers with a *p*-value cutoff of 0.01 and log2FC cutoff of 1 from the Gene Expression Profiling and Interactive Analysis (GEPIA) database. **(C)** Dot plot showing the expression profile of TRPM2 across multiple cancers and paired normal tissues. Each *dot* represents the expression of samples from the GEPIA database. *p < 0.05, **p < 0.01, ***p < 0.001.

GEPIA (http://gepia.cancer-pku.cn/) is an interactive web server for analyzing the RNA sequencing expression data of 9,736 tumors and 8,587 normal samples from TCGA and the Genotype-Tissue Expression (GTEx) project, respectively (Tang et al., 2017). The GEPIA database was utilized to evaluate the tumor/normal differential expression levels of TRPM2 and to conduct patient survival analysis based on the expression levels of TRPM2 across various cancer types. The GEPIA database utilizes log2(TPM + 1) for log scale with a |Log2FC| cutoff of 1 and a *p*-value cutoff of 0.01 in determining the differential expression of TRPM2, shown in Figure 1B.

### Kaplan–Meier Plotter Database Analysis

The Kaplan–Meier plotter (http://kmplot.com/analysis/) can assess the effect of 54 k genes (mRNA, miRNA, and protein) on survival in 21 cancer types and discover and validate survival biomarkers (Gyorffy, 2021). The databases Gene Expression Omnibus (GEO), European Genome–Phenome Archive (EGA), and TCGA were used to assess the correlation between clinical outcomes and TRPM2 expression in different cancers and between TRPM2 expression and immune cell infiltration. We also calculated the hazard ratios (HRs) of 95% confidence intervals (CIs) and the log-rank *p*-value.

### TISIDB

The TISIDB database (http://cis.hku.hk/TISIDB/index.php) is a portal for analyzing tumor and immune cell interactions that integrates multiple heterogeneous data types (Ru et al., 2019). Next, we analyzed the correlation between TRPM2 expression and clinical features, such as overall survival (OS), cancer stage, and cancer grade. Heatmap and dot plots showing the Spearman’s correlations between the expression of TRPM2 and the abundance of tumor immune-infiltrating cells across diverse cancer types were also generated using the TISIDB database.

### UALCAN Database Analysis

The UALCAN database (http://ualcan.path.uab.edu/index.html) is available for online analysis of cancer omics data (TCGA, MET500, and CPTAC). This database allows users to identify biomarkers, perform pan-cancer gene expression analysis, obtain patient survival information, and analyze epigenetic regulation of gene expression (Chandrashekar et al., 2017). We used this database to validate the results of the correlation between TRPM2 expression and clinical features in the TISIDB database. A *p* < 0.05 indicated statistically significant differences.

### Statistical Analysis

The expression of TRPM2 was analyzed *via* the TIMER and GEPIA databases. The correlation between TRPM2 expression and survival prognosis, including OS, disease-free survival (DFS), and relapse-free survival (RFS) in pan-cancer, were analyzed in the GEPIA database and the Kaplan–Meier plotter platform. To compare the survival curves, we used the log-rank test to calculate the HRs, 95% CIs, and *p*-values in the Kaplan–Meier plotter and GEPIA. We used Spearman’s correlation analysis to evaluate the correlation between gene expression and immune cell infiltration in the TIMER and TISIDB databases. A *p* < 0.05 was considered statistically significant.

## Results

### *TRPM2* mRNA Level in Different Cancers

The mRNA level *TRPM2* was analyzed in the TIMER (Figure 1A) and GEPIA databases (Figures 1B, C). The results showed that TRPM2 was highly expressed in a majority of cancers (Figure 1A). We also used the GEPIA database to validate the findings in the TIMER database and found that, compared to the corresponding normal tissues, the mRNA level of *TRPM2* was significantly higher in most human tumors, including bladder urothelial carcinoma (BLCA), breast invasive carcinoma (BRCA), cervical squamous cell carcinoma and endocervical adenocarcinoma (CESC), cholangiocarcinoma (CHOL), colon adenocarcinoma (COAD), esophageal carcinoma (ESCA), head and neck squamous cell carcinoma (HNSC), kidney renal clear cell carcinoma (KIRC), kidney renal papillary cell carcinoma (KIRP), acute myeloid leukemia (LAML), ovarian serous cystadenocarcinoma (OV), pancreatic adenocarcinoma (PAAD), rectum adenocarcinoma (READ), skin cutaneous melanoma (SKCM), stomach adenocarcinoma (STAD), uterine corpus endometrial carcinoma (UCEC), and uterine carcinosarcoma (UCS) (Figures 1B, C). Moreover, the level of TRPM2 was significantly lower in glioblastoma multiforme (GBM) and brain lower-grade glioma (LGG) than that in normal tissue, while no significant differences were detected in sarcoma (SARC), testicular germ cell tumors (TGCT), adrenocortical carcinoma (ACC), diffuse large B-cell lymphoma (DLBC), kidney chromophobe (KICH), liver hepatocellular carcinoma (LIHC), lung adenocarcinoma (LUAD), pheochromocytoma and paraganglioma (PCPG), thyroid carcinoma (THCA), and thymoma (THYM) (Figures 1B, C).

### Prognostic Significance of TRPM2 Expression in Various Cancers

**FIGURE 2:**
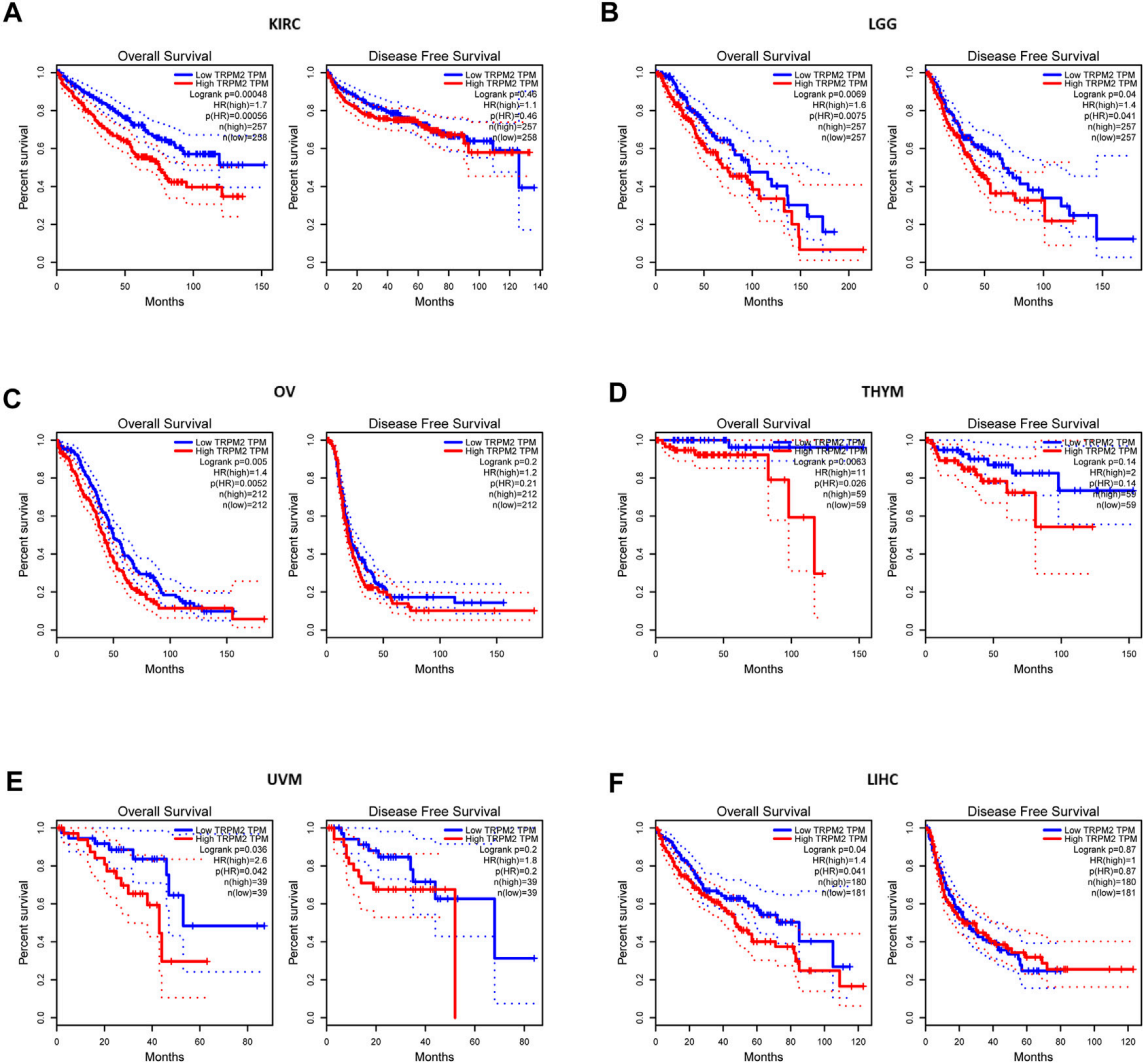
Comparison of the survival curves of TRPM2 expression in different cancers in the Gene Expression Profiling and Interactive Analysis (GEPIA) database. Correlation curves between the level of TRPM2 and overall survival (OS) and disease-free survival (DFS) of kidney renal clear cell carcinoma (KIRC) **(A)**, lower-grade glioma (LGG) **(B)**, ovarian serous cystadenocarcinoma (OV) **(C)**, thymoma (THYM) **(D)**, uveal melanoma (UVM) **(E)**, and liver hepatocellular carcinoma (LIHC) **(F)** patients. *Red curve* represents patients with high expression of TRPM2. *Blue curve* represents patients with low expression of TRPM2.

Next, we investigated the prognostic value of TRPM2 in different cancers using GEPIA and the Kaplan–Meier plotter platform. In GEPIA, the Kaplan–Meier plots revealed that a high TRPM2 level was associated with a short OS and/or DFS in cancers, including KIRC (OS: HR = 1.7, *p* = 0.00048; DFS: HR = 1.1, *p* = 0.46), LGG (OS: HR = 1.6, *p* = 0.0069; DFS: HR = 1.4, *p* = 0.04), OV (OS: HR = 1.4, *p* = 0.005; DFS: HR = 1.2, *p* = 0.2), THYM (OS: HR = 11, *p* = 0.0063; DFS: HR = 2, *p* = 0.14), uveal melanoma (UVM) (OS: HR = 2.6, *p* = 0.036; DFS: HR = 1.8, *p* = 0.2), and LIHC (OS: HR = 1.4, *p* = 0.04; DFS: HR = 1, *p* = 0.87) (Figure 2).

Furthermore, using the Kaplan–Meier plotter, we found that the expression of TRPM2 was negatively correlated with prognosis in KIRC (OS: HR = 1.7, *p* = 0.00056; RFS: HR = 0.38, *p* = 0.19), OV (OS: HR = 1.51, *p* = 0.0024; RFS: HR = 0.81, *p* = 0.32), and KIRP (OS: HR = 0.4, *p* = 0.015; RFS: HR = 2.59, *p* = 0.0098) (Supplementary Figure S1). These findings indicated that TRPM2 was a prognostic factor for short OS in KIRC. In contrast, the expression level of TRPM2 was positively correlated with the prognosis of BLCA, READ, UCES, THCA, and STAD, as predicted by GEPIA and the Kaplan–Meier plotter (Supplementary Figure S2).

### Clinical Characteristics of TRPM2 in Cancers

**FIGURE 3:**
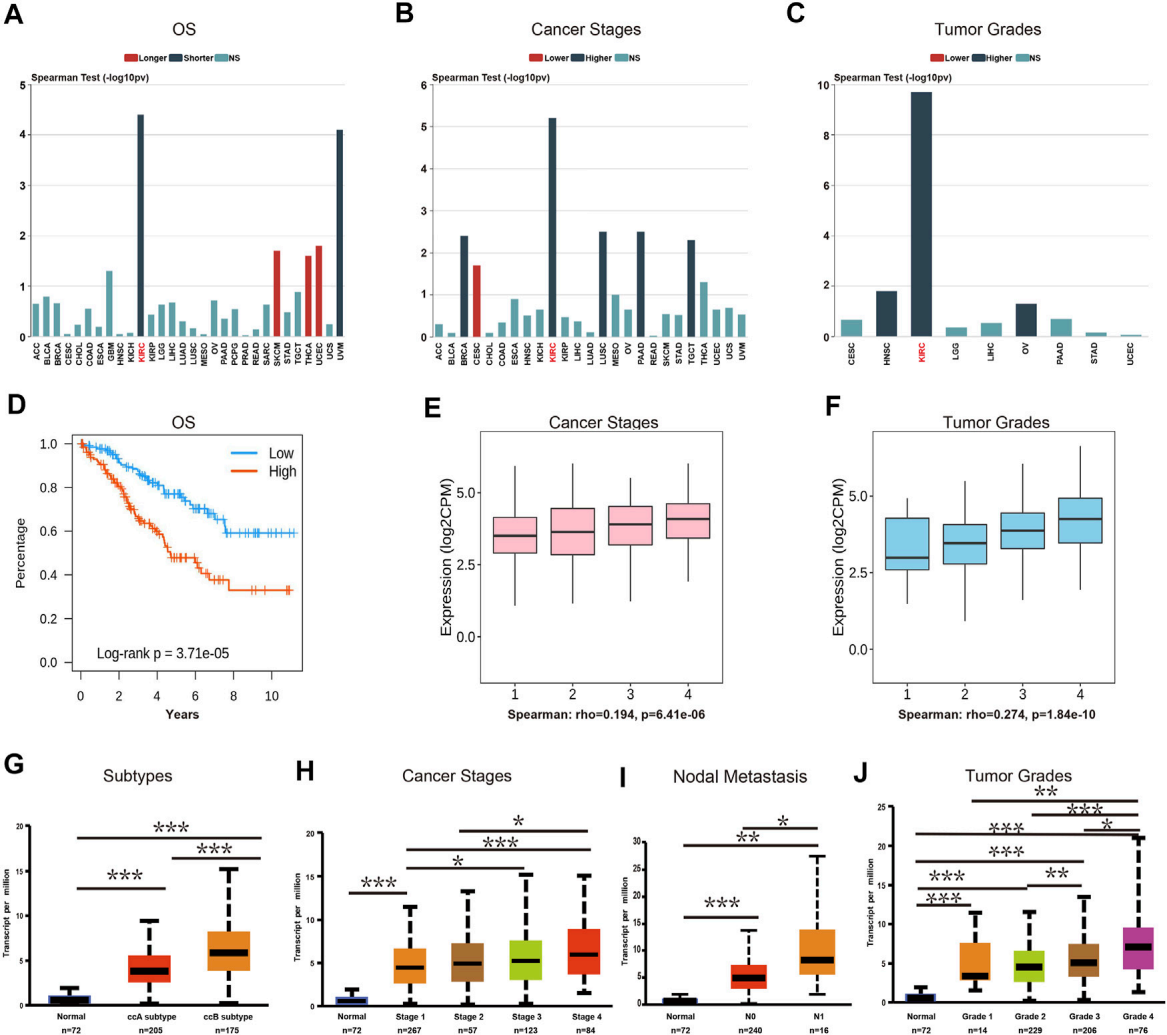
The expression level of TRPM2 was correlated with subtype, cancer stage, nodal metastasis, and tumor grade in kidney renal clear cell carcinoma (KIRC). **(A**–**C)** Correlation between the mRNA level of *TRPM2* and patients’ overall survival (OS) **(A)**, cancer stage **(B)**, and cancer grade **(C)** in multiple cancers using the TISIDB database. **(D**–**F)** Associations between the expression level of TRPM2 and OS **(D)**, stage **(E)**, and grade **(F)** of KIRC. **(G**–**J)** UALCAN analysis of the associations between TRPM2 expression and subtype **(G)**, cancer stage **(H)**, nodal metastasis **(I)**, and tumor grade **(J)** in KIRC. Data in **(A**–**F)** were derived from the TISIDB database (an integrated repository portal for tumor–immune system interactions). Data in **(G**–**J)** were analyzed using the UALCAN database. *N0*, metastases in one to three axillary lymph nodes; *N1*, metastases in one to three axillary lymph nodes. *p < 0.05, **p < 0.01, ***p < 0.001.

In order to examine the association between TRPM2 and clinical features, we assessed the multiple clinical prognostic values of TRPM2 in various cancers using the TIDISB database. Among the human cancers, KIRC was the most correlated cancer with regard to OS (Figure 3A), stage (Figure 3B), and tumor grade (Figure 3C). The association plots derived from the 533 KIRC cases in TCGA showed that the upregulation of TRPM2 expression was significantly associated with shorter OS, poor pathological stage, and tumor grade in KIRC (Figure 3D–F, respectively). Using the UALCAN database, we further validated the finding that the expression level of TRPM2 was negatively correlated with the prognosis of KIRC patients, in consideration of the tumor subtype (Figure 3G), cancer stage (Figure 3H), nodal metastasis (Figure 3I), and tumor grade (Figure 3J). The results demonstrated a significant correlation between high TRPM2 levels and these adverse clinicopathological parameters, which was consistent with those described above (Figure 2A). Therefore, we mainly focused on the function of TRPM2 in KIRC to examine its prognostic performance, clinical significance, and mechanism.

**FIGURE 4:**
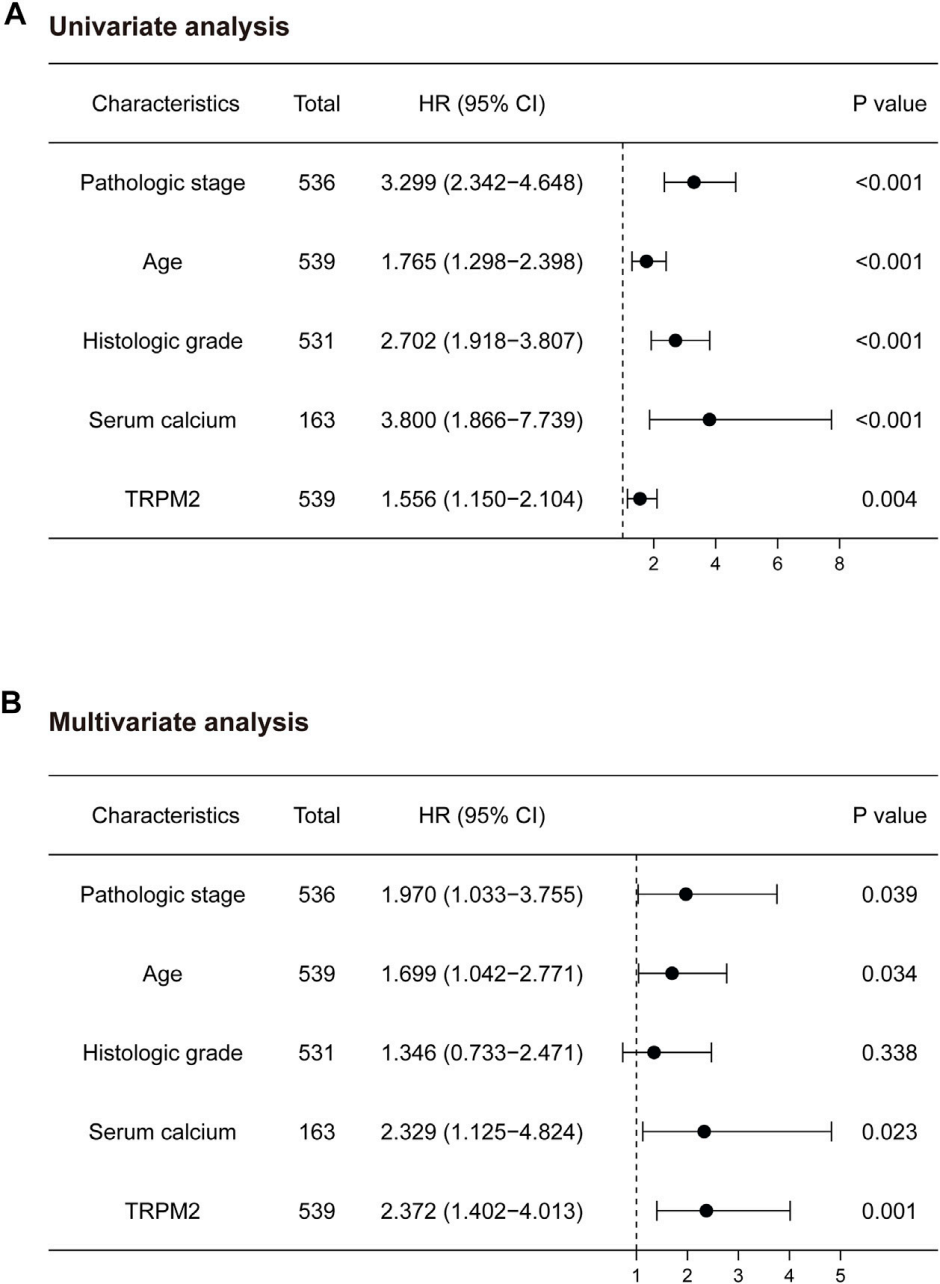
Univariate and multivariate analyses of the factors associated with the overall survival (OS) of kidney renal clear cell carcinoma (KIRC) patients from The Cancer Genome Atlas (TCGA) database. Forest plots display the prognostic values of factors associated with the OS of KIRC patients. HR and *p*-values were calculated using univariate **(A)** or multivariate **(B)** Cox proportional hazards regression. *Blue circles* represent the hazard ratio (HR).

To elucidate whether TRPM2 is an independent risk factor for clinical outcomes of KIRC patients, univariate and multivariate Cox analyses were performed. In the univariate Cox analysis, the pathological stage, age, histologic grade, serum calcium, and TRPM2 expression were significantly correlated with OS (*p* < 0.001, *p* < 0.001, *p* < 0.001, *p* < 0.001, and *p* = 0.005, respectively) (Figure 4A). To exclude the confounder effect, the significant prognostic factors detected in the univariate analysis (Figure 4A) were evaluated further in the multivariate analysis (Figure 4B). Strikingly, multivariate Cox analysis revealed that the pathological stage, age, serum calcium, and TRPM2 expression were independent risk factors for OS (*p* = 0.039, *p* = 0.034, *p* = 0.023, and *p* = 0.001, respectively) (Figure 4B) of KIRC patients. Since calcium influx plays a critical role in tumor occurrence and development through TRPM2 channels, we considered serum calcium for the above Cox analysis. Interestingly, both univariate and multivariate analyses verified that serum calcium was an independent risk factor for the OS of KIRC patients (HR = 2.329, 95% CI = 1.125–4.824, *p* = 0.023) (Figure 4B). Based on the clinicopathological features and TRPM2, we also constructed a nomogram to predict the 1-, 5-, and 10-year OS rates using the Cox regression algorithm (Supplementary Figure S3).

### TRPM2 Expression Was Correlated With Immune Infiltration in Kidney Renal Clear Cell Carcinoma

**FIGURE 5:**
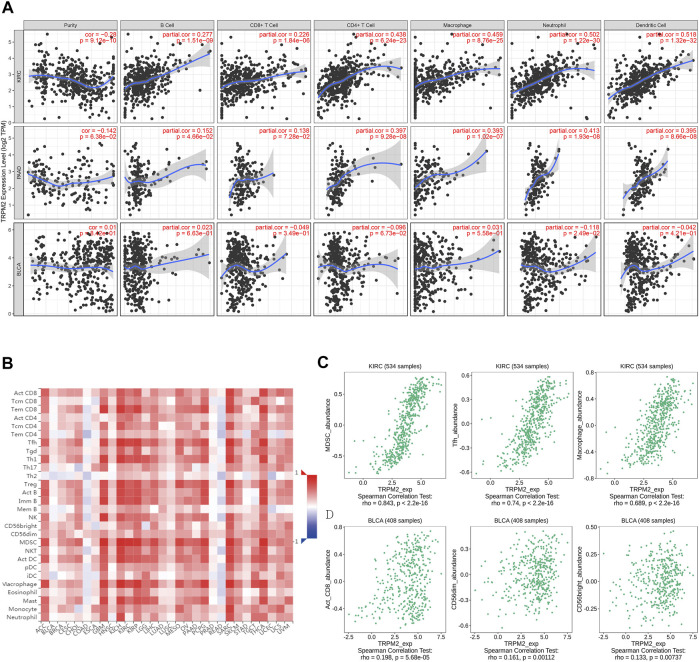
Correlation analysis between the expression level of TRPM2 and immune cell infiltration. **(A)** Correlation between the expression level of TRPM2 and tumor purity, infiltrating levels of B cells, CD8^+^ T cells, CD4^+^ T cells, macrophages, neutrophils, and dendritic cells in kidney renal clear cell carcinoma (KIRC), pancreatic adenocarcinoma (PAAD), and bladder urothelial carcinoma (BLCA) displayed using the TIMER 1.0 database. **(B)** Correlations between the infiltration level of tumor immune cells and the expression level of TRPM2 across human cancers in the TISIDB database. **(C**, **D)** Top 3 types of TRPM2-correlated immune cells in KIRC **(C)** and BLCA **(D)** using the TISIDB database.

The features of TIME are emerging as critical biomarker in predicting the prognosis of patients and the efficacy of treatment. Recent studies have suggested a high immune infiltration level in KIRC with the highest overall T cells, CD8^+^ T cells, T helper 1 (Th1) cell, dendritic cells, neutrophils, and cytotoxic cells (Vuong et al., 2019). Therefore, we analyzed the correlation of the level of TRPM2 with the immune infiltration level in various cancer types. The results showed that the expression of TRPM2 was significantly positively correlated with B cells (*r* = 0.277, *p* = 1.51e−09), CD8^+^ T cells (*r* = 0.226, *p* = 1.84e−06), CD4^+^ T cells (*r* = 0.438, *p* = 6.24e−23), macrophages (*r* = 0.459, *p* = 8.76e−25), neutrophils (*r* = 0.502, *p* = 1.22e−30), and dendritic cells (*r* = 0.518, *p* = 1.32e−32) in KIRC (Figure 5A). Moreover, the level of TRPM2 showed a positive correlation with the infiltration of B cells (*r* = 0.152, *p* = 4.66e−02), CD8^+^ T cells (*r* = 0.138, *p* = 7.28e−02), CD4^+^ T cells (*r* = 0.397, *p* = 9.28e−08), macrophages (*r* = 0.393, *p* = 1.02e−07), neutrophils (*r* = 0.413, *p* = 1.93e−08), and dendritic cells (*r* = 0.395, *p* = 8.66e−08) in PAAD (Figure 5A). However, TRPM2 was not correlated with B cells (*r* = 0.023, *p* = 6.63e−01), CD8^+^ T cells (*r* = −0.049, *p* = 3.49e−01), CD4^+^ T cells (*r* = −0.096, *p* = 6.73e−02), macrophages (*r* = 0.031, *p* = 5.58e−01), neutrophils (*r* = −0.118, *p* = 2.49e−02), and dendritic cells (*r* = −0.042, *p* = 4.21e−01) in BLCA (Figure 5A).

Then, the TISIDB database was used to further explore the correlations between the level of TRPM2 and the 28 tumor immune-infiltrating cell subtypes. The results showed that the level of TRPM2 was associated with 25 immune cell subtypes in KIRC (Figure 5B). Notably, myeloid-derived suppressor cells (MDSCs) (*r* = 0.843, *p* < 2.2e−16), T follicular helper (Tfh) cells (*r* = 0.74, *p* < 2.2e−16), and macrophages (*r* = 0.689, *p* < 2.2e−16) displayed relative strong correlation with TRPM2 expression in KIRC (Figure 5C). Others, such as immature B cells (*r* = 0.667, *p* < 2.2e−16), Th1 (*r* = 0.66, *p* < 2.2e−16), effector memory CD8^+^ T cells (*r* = 0.637, *p* < 2.2e−16), activated dendritic cells (*r* = 0.641, *p* < 2.2e−16), activated CD4 T cells (*r* = 0.546, *p* < 2.2e−16), Tregs (*r* = 0.617, *p* < 2.2e−16), activated B cells (*r* = 0.64, *p* < 2.2e−16), natural killer (NK) cells (*r* = 0.532, *p* < 2.2e−16), NK T cells (*r* = 0.584, *p* < 2.2e−16), and mast cells (*r* = 0.539, *p* < 2.2e−16), were moderately correlated with TRPM2 (Figure 5B). In addition, TRPM2 showed a weak correlation with immune cells of activated CD8^+^ cells (*r* = 0.198, *p* = 5.68e−05), CD56dim (*r* = 0.161, *p* = 0.00112), and CD56bright (*r* = 0.133, *p* = 0.00737) in BLCA (Figure 5D). In addition, the correlation between the level of TRPM2 and the degree of immune infiltration in various cancers were analyzed using the TIMER database and displayed as a heatmap (Supplementary Figure S4). These findings strongly indicated that TRPM2 serves as a major tumor immune infiltration regulator in KIRC.

### TRPM2 Expression Was Correlated With Immune Cell Type Markers

We assessed the correlation between the expression of TRPM2 and the level of tumor-infiltrating immune cell gene markers in KIRC and BLCA using the TIMER database. The results showed that the level of TRPM2 in KIRC tissues was strongly associated with immune markers of B cells, CD8^+^ T cells, dendritic cells, M1/M2 macrophages, monocytes, neutrophils, general T cells, T-cell exhaustion, tumor-associated macrophages (TAMs), Th1, Th2, and Tregs, but not with NK, Tfh, and Th17 cells (Table 1). A major factor determining tumor progression over time is the overall proportion and property of T cells within the TIME (Binnewies et al., 2018). Notably, the level of TRPM2 was significantly correlated with various subtypes of T cells based on cell markers, including CD8^+^ T-cell markers (CD8A and CD8B), T-cell (general) markers (CD3D, CD3E, and CD2), exhausted T-cell markers (CTLA4, GZMB, LAG-3, and PDCD 1), Th1 markers (TBX21, STAT4, STAT1, and IFNG), Th2 markers (GATA3 and STAT5A), Treg markers (FOXP3 and CCR8), and with neutrophil markers (ITGAM and CCR7), dendritic cell markers (ITGAX, HLA-DPA1, HLA-DRA, and HLA-DPB1), and B-cell markers (CD79A and CD19) in KIRC (Table 1). Also, a significant correlation was established between the level of TRPM2 and the expressions of marker genes in different subsets of macrophages, including M1 macrophage markers (IRF5), M2 macrophage markers (MS4A4A, VSIG4, and CD163), and TAMs (IL10 and CD68), in KIRC (Table 1). However, only nine immune cell marker genes were significantly associated with the levels of TRPM2 in BLCA. Furthermore, the expression of TRPM2 was not markedly related to most marker genes of CD8^+^ T cells, NK cells, Th2, and Th17 cells in BLCA. These findings revealed that TRPM2 is involved in the regulation of tumor immune infiltration in KIRC.

**TABLE 1:** Correlation analysis between TRPM2 and the gene markers of different immune cells in kidney renal clear cell carcinoma (KIRC) and bladder urothelial carcinoma (BLCA) in TIMER 2.0 database.

Description	Gene markers	KIRC				BLCA			
		None		Purity		None		Purity	
		cor	*p*-value	cor	*p*-value	cor	*p*-value	cor	*p*-value
B cells	CD19	0.485744546488383	6.59706240957069E−33	0.44912716101394	2.88405876255151E−24	0.140977998	0.004328931	0.142818690042179	0.006060331222284
	CD79A	0.457302777544265	6.65990580429951E−29	0.416277125388441	9.54975490987652E−21	0.028532928579865	0.565503885756219	0.007049091073815	0.892796379854096
CD8^+^ T cells	CD8A	0.5128382486411	4.35603735096699E−37	0.447274430400181	4.66558704926754E−24	0.001688327917082	0.972878786994711	-0.016488961057195	0.752565440794958
	CD8B	0.520930818273473	2.0703319159061E−38	0.468860929168814	1.42505157022276E−26	0.064160270945273	0.19589670161921	0.06545123281899	0.210335172871856
Dendritic cells	ITGAX	0.672734376800159	1.72298510762689E−71	0.658568357837	1.18303891796421E−58	0.285082745	5.4E−10	0.112053862900684	0.031632412032205
	NRP1	−0.081939468348264	0.058696713329523	−0.132860445073218	0.004269006458162	−0.075536927104492	0.127691935446066	−0.074884351590772	0.151670056459206
	CD1C	0.247420777178183	7.08118613449626E−09	0.203327892125407	1.08337400911216E−05	−0.130116103647726	0.008504688518478	−0.139836245199018	0.00721810684817
	HLA-DPA1	0.528197321944636	1.25123152666063E−39	0.486142394778355	1.01701661178129E−28	-0.004569731674141	0.926681083160627	−0.015851867616873	0.761831903602285
	HLA-DRA	0.548021003005431	4.15109691526204E−43	0.524285169619602	6.50942764966405E−34	−0.012360016249858	0.803435328672719	−0.029577471272472	0.571674074983743
	HLA-DQB1	0.400042232509181	6.67449268929836E−22	0.358203141697989	2.10687509054187E−15	0.023575016632827	0.634931667183906	0.024273344932363	0.642556231032367
	HLA-DPB1	0.617375673595729	2.58666966272962E−57	0.597544202912104	5.86148664455409E−46	0.027258752574709	0.582996823501823	0.021973959079056	0.674375676816137
M1 macrophages	PTGS2	0.037605477752189	0.38623900370777	−0.017596499080294	0.706310114744905	−0.112104938356949	0.023536974899908	−0.125382496185421	0.016102910629493
	IRF5	0.478350757545123	7.88268281521714E−32	0.478440402122546	9.52979179671435E−28	0.054390997610448	0.273041523951748	0.074060259806906	0.156238512533157
	NOS2	−0.037207007014409	0.391295339242913	−0.084574963783569	0.069643377197893	−0.059642341895096	0.229328477825857	−0.084865563114879	0.104077645675281
M2 macrophage	MS4A4A	0.501365176332907	2.8513130424673E−35	0.461239207589862	1.15444183256125E−25	0.065892229093315	0.184072983067615	0.075170477846244	0.150107804228774
	VSIG4	0.442732199276045	5.44944257580824E−10	0.583721896295131	1.89508299387773E−43	0.058151416537689	0.241201361661768	0.061333802234553	0.240519705508872
	CD163	0.445254149612406	2.5675001741038E−27	0.428135271738487	5.66884711210626E−22	0.073567755984626	0.137955558194911	0.087562832461627	0.093492685812632
Monocytes	CSF1R	0.708784169553575	1.64211189013342E−82	0.702590511184529	7.90420170002513E−70	0.022864647490321	0.645167068936561	0.026114346446434	0.617539249463632
	CD86	0.653617502882632	2.94706197567482E−66	0.625840745086099	1.7050962799574E−51	0.02235685046937	0.652525115203618	0.020070762917123	0.701161692760728
Natural killer cells	KIR2DS4	0.076401647629758	0.078017878595369	0.054080383206576	0.246516980483003	0.042657575840631	0.390126226737769	0.050559105020527	0.333438628679095
	KIR3DL3	0.095437864235261	0.027579783332291	0.072444882739474	0.120357162235272	−0.048935684169912	0.324127922668039	−0.03751383746786	0.473099875571284
	KIR3DL2	0.177070070590737	3.94101995361704E−05	0.15795979372866	0.000664735698086	−0.000184420482565	0.997036917010056	−0.008830540246802	0.865933413302723
	KIR3DL1	0.023663708379846	0.585675907779733	0.023388058536703	0.616463943875371	0.048293253974367	0.330527979940115	0.020778822480965	0.691150862188917
	KIR2DL4	0.285210021226047	1.96004995245962E−11	0.251592384250719	4.36096806222996E−08	−0.021905197378405	0.659098083782204	−0.023664457678256	0.650921806220696
	KIR2DL3	0.065552202481811	0.130670510860898	0.065263515546813	0.161826597573493	−0.006473403047136	0.89628435668632	−0.019255995617379	0.712745373013371
	KIR2DL1	0.045718827830429	0.292077988638567	0.006895141840321	0.882623094371932	0.022290295485346	0.653492027390003	−0.004727315608632	0.927987137532812
Neutrophils	CCR7	0.46040635813648	2.53852711953024E−29	0.427082760030601	7.31696606426214E−22	0.158652821924251	0.001303605610734	0.145494522867536	0.005166441866428
	ITGAM	0.629108092654827	4.43710764402331E−60	0.620813407868791	1.80647498970577E−50	0.062168472616264	0.210168839486813	0.068246055701041	0.191465580407394
	CEACAM8	0.006469263321554	0.881549329595812	0.023823458463915	0.609914807860632	0.017942314893252	0.717849652621743	0.021580233531224	0.679884806244015
T cells (general)	CD3D	0.600447918982057	1.59641932229757E−53	0.544329148781585	6.44150616326556E−37	−0.004941039039642	0.920742479293529	−0.022464307534244	0.667538830876612
	CD3E	0.588641638602304	5.196354456764E−51	0.528730607955272	1.46020077851106E−34	0.533398247322298	4.16384968594744E−07	-0.014812173867	0.777026889904191
	CD2	0.590749380752671	1.88242323399633E−51	0.532577185260379	3.93478610416807E−35	0.005948416285955	0.904653824351816	−0.006502267751281	0.901068182765454
T-cell exhaustion	CTLA4	0.577037778923657	1.22042917827904E−48	0.525070144333357	5.00744589565522E−34	0.004841476175388	0.922334446485858	−0.008717336352999	0.867636199906032
	LAG3	0.597396356695186	7.28732596613059E−53	0.545239226974596	4.65293857029099E−37	0.01351740121358	0.785458352092061	−0.000270781344593	0.995869517714635
	HAVCR2	0.149944424705605	0.000514169572285	0.100701835949916	0.030634521790755	0.063065775041768	0.203649092130114	0.079995549099584	0.125567908730434
	GZMB	0.346366243934104	1.81135611072699E−16	0.277153262122229	1.41674172033825E−09	0.01147155017657	0.817306531206087	−0.002851366645297	0.956526675625948
	PDCD1	0.62712905071317	1.3240936915026E−59	0.582520780290401	3.0907491014941E−43	0.060902121363906	0.219624182053375	0.05827664711153	0.26481419847595
TAM	CCL2	0.120161593039727	0.005474452875158	0.067695083703023	0.1467258006677	0.095047269603777	0.055071034467237	0.082640422910232	0.113505143186069
	IL10	0.461198154330114	1.98163949979899E−29	0.408255160726959	6.06051054543883E−20	0.047981954299883	0.333658335828777	0.039726300626898	0.447380177972515
	CD68	0.439445337144535	1.41851318788575E−26	0.452729634130137	1.12235853416841E−24	−0.087095581702116	0.078885856096736	−0.08802346577886	0.091774152515539
Tfh	BCL6	0.082214544669031	0.05785089642678	0.07936525877062	0.0887359049861	−0.083821757414992	0.090856141293264	−0.086148913096961	0.098928768161036
	IL21	0.178368162335664	3.44984612377128E−05	0.153886966409005	0.00091679496478	0.058900016965508	0.235187103224401	0.084777977606767	0.007488256895318
Th1	TBX21	0.379466735133681	1.06599724819939E−19	0.338165366016663	8.52428962036312E−14	0.042594355695284	0.390829700141107	0.042739980523245	0.413654597911359
	STAT4	0.471209369213796	8.17665799965634E−31	0.41046035332717	3.66483277736669E−20	−0.029036043430605	0.558666578186851	−0.041381035393888	0.428673087894851
	STAT1	0.411028422402828	3.82990322371071E−23	0.35635649139502	2.99624194477578E−15	−0.053329906793009	0.282521928292724	-0.064960177817876	0.213784470711136
	IFNG	0.503881560765259	1.15513515829342E−35	0.443514596853995	1.2272300844711E−23	0.064020259406744	0.19687621727213	0.054304326081059	0.29882318260954
	IL13	0.151621535765502	0.000443803952566	0.124446979908192	0.007469751279614	−0.069600830913532	0.160540278343667	−0.085250125229893	0.102512984353924
Th2	GATA3	0.230655154471105	7.22960523715275E−08	0.184835656387673	6.54448666852358E−05	0.090037677072457	0.069250237361316	0.084038198421818	0.107507961669913
	STAT6	0.096224977149154	0.026319309440385	0.140810824207718	0.002443596368969	−0.000299306942759	0.995191054811795	0.015929883016694	0.760695317975642
	STAT5A	0.582666321161584	8.88023115804845E−50	0.582666321161584	8.88023115804845E−50	0.138113368865284	0.005196415833676	0.138113368865284	0.005196415833676
Th17	STAT3	0.087808232077976	0.042727243984499	0.063511353635169	0.173412869154382	−0.152736209209546	0.001975930647598	-0.166664763797919	0.001333001185945
	IL17A	0.053140929738898	0.220635940856933	0.022115908580037	0.635772717650214	−0.039436809580501	0.426933715585849	−0.08159297246896	0.118168873327965
Tregs	FOXP3	0.570300340798301	2.63107826051956E−47	0.516414746796316	8.70043412068396E−33	−0.03236304907375	0.51448714964267	−0.047173906207336	0.366857062325231
	CCR8	0.459998229150614	2.88344749611744E−29	0.407210155416581	7.68185883379204E−20	−0.005572301802663	0.910656483651222	−0.021330064238666	0.683394138902167
	STAT5B	−0.094923644968207	0.028430999438215	−0.085420498996765	0.066887822464905	−0.054804532379945	0.269406149621036	−0.060552484249918	0.246574520991994
	TGFB1	0.160199699775916	0.000204148701863	0.118668892667721	0.010771420491892	−0.09999254824657	0.043527210629257	−0.087539337378134	0.093581025920307

Purity and none are the Spearman’s rho between TRPM2 and immune infiltration level after or without purity adjustment, respectively.

*TAMs*, tumor-associated macrophages; *cor*, correlation.

*p < 0.01, **p < 0.001, ***p < 0.0001.

### Role of TRPM2 Level in Prognosis Prediction of KIRC Based on Different Enriched Immune Cell Cohorts

**FIGURE 6:**
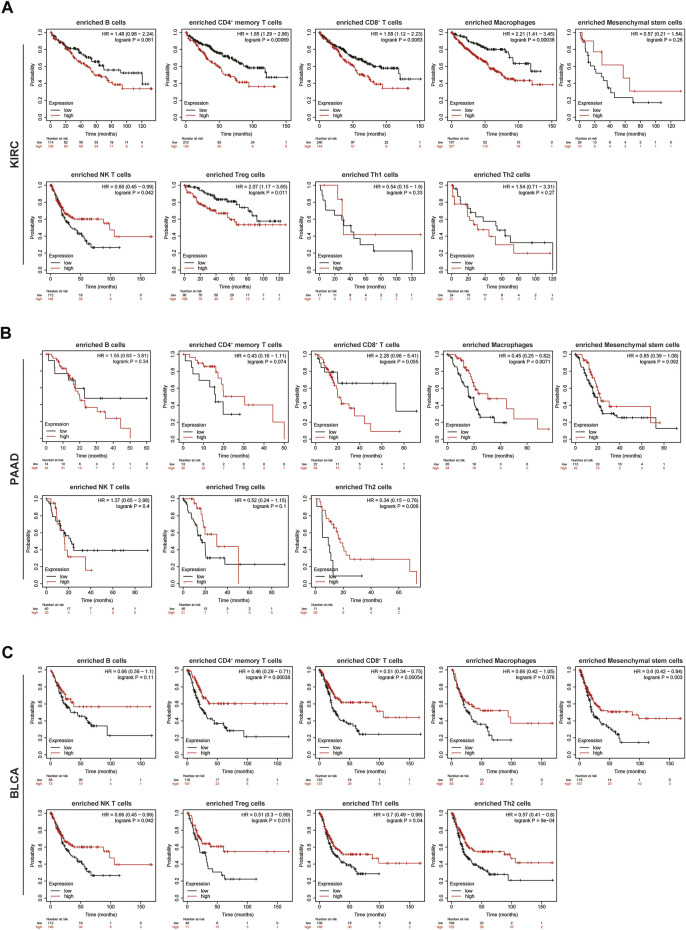
Comparison of the Kaplan–Meier survival curves of overall survival (OS) showing the expression of TRPM2 in kidney renal clear cell carcinoma (KIRC), pancreatic adenocarcinoma (PAAD), and bladder urothelial carcinoma (BLCA) based on the differentially enriched immune cell cohorts. The association of the level of TRPM2 with OS was shown in KIRC **(A)**, PAAD **(B)**, and BLCA **(C)** patients based on the differentially enriched immune cell subgroups in the Kaplan–Meier plotter.

TRPM2 is involved in the regulation of tumor immune infiltration in KIRC (Figure 5 and Table 1). Moreover, upregulated TRPM2 indicated poor prognosis in KIRC/PAAD patients, but better prognosis in BLCA patients. Thus, we hypothesized that TRPM2 may affect the prognosis of patients partially through the regulation of immune infiltration. Analysis of the Kaplan–Meier plotter revealed that the level of TRPM2 was associated with OS in KIRC, PAAD, and BLCA patients based on the enrichment of different immune cells. In KIRC, a high TRPM2 level in enriched CD4^+^ memory T cells, CD8^+^ T cells, macrophages, and Tregs indicated poor prognosis of patients with KIRC (Figure 6A). However, no significant correlation was established between the expression of TRPM2 and OS in enriched B cells, mesenchymal stem cells, Th1 cells, and Th2 cells (Figure 6A). On the other hand, in PAAD, a high expression of TRPM2 was related to poor prognosis only in CD8^+^ T cells and enriched B cells, but showed no statistically significant difference (Figure 6B). The clinical samples for Th1 cell enrichment were too few to analyze in PAAD. In BLCA, low-TRPM2 expression groups showed short OS in enriched CD4^+^ memory T cells, CD8^+^ T cells, Tregs, mesenchymal stem cells, NK T cells, Th2 cells, Th1 cells, B cells, and macrophages (Figure 6C). The above results suggested that TRPM2 may affect the prognosis of patients by potentially regulating the infiltration of immune cells in KIRC.

### Enrichment of TRPM2-Correlated Genes in KIRC Patients by Gene Ontology/Kyoto Encyclopedia of Genes and Genomes Analysis

**FIGURE 7:**
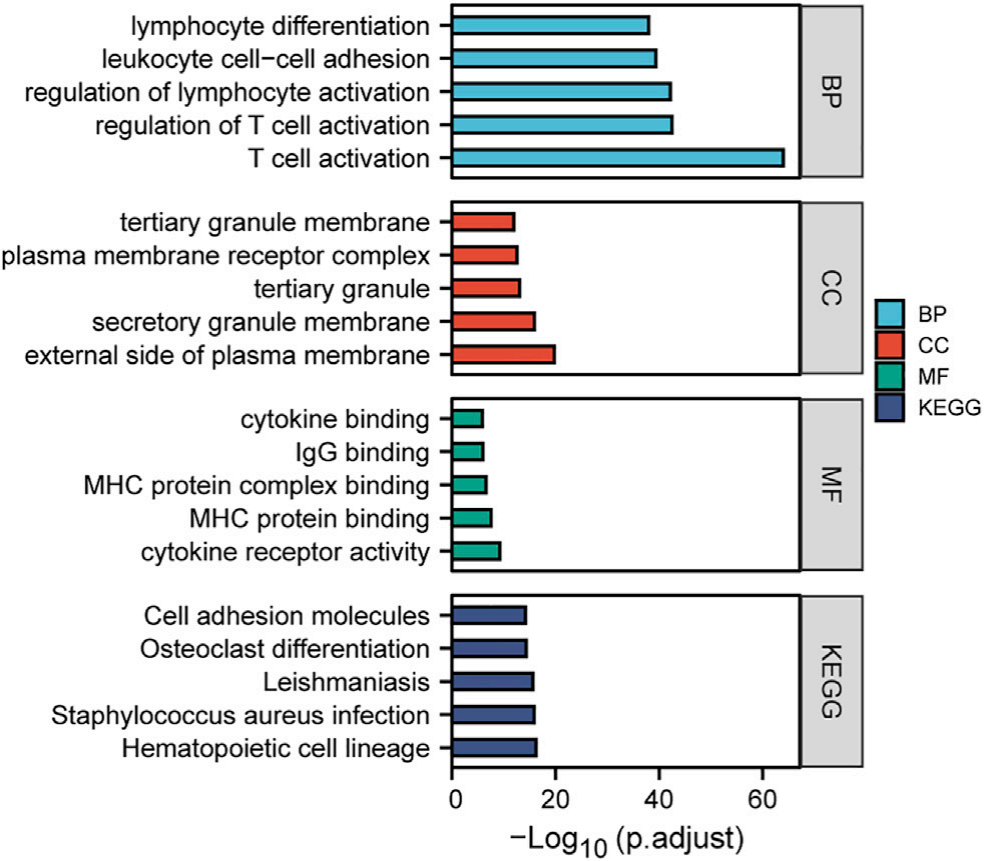
Gene Ontology (GO) and Kyoto Encyclopedia of Genes and Genomes (KEGG) enrichment analyses of the TRPM2-related genes in kidney renal clear cell carcinoma (KIRC). Gene expression was calculated using Pearson’s correlation analysis based on KIRC RNA-seq data from The Cancer Genome Atlas (TCGA), and then the TRPM2-related genes (|*r*| > 0.5 and *p* < 0.05) in KIRC were subjected to GO classification and KEGG pathway enrichment analysis (https://www.xiantao.love/).

To gain in-depth insights into the molecular mechanism of TRPM2 as a prognostic marker in KIRC, the gene expression correlation was calculated with Pearson’s correlation analysis to reveal the genes associated with TRPM2 in KIRC patients. Subsequently, we conducted GO and KEGG analysis of the TRPM2-related genes in KIRC. Pearson’s correlation coefficients between TRPM2 and the genes of interest were calculated, and only genes with |*r*| > 0.5 and *p* < 0.05 were subjected to GO classification and KEGG pathway enrichment. The top 5 GO categories are shown in Figure 7. In KIRC, the above genes were enriched in several biological processes, such as T-cell activation, regulation of T-cell activation, lymphocyte activation, leukocyte cell–cell adhesion, and lymphocyte differentiation. For molecular function (MF), these genes were mainly associated with cytokine receptor activity, MHC protein binding, MHC protein complex binding, IgG binding, and cytokine binding. Figure 7 also shows the most significant KEGG pathways, namely, hematopoietic cell lineage, *Staphylococcus aureus* infection, leishmaniasis, osteoclast differentiation, and cell adhesion molecules. In addition, GO cell component (CC) analysis indicated that TRPM2 plays a key role in the regulation of T-cell activation on the external side of the plasma membrane, secretory granule membrane, tertiary granule membrane, plasma membrane receptor complex, and tertiary granule membrane in KIRC.

Conversely, in BLCA, TRPM2-correlated genes (|*r*| > 0.5 and *p* < 0.05) failed to enrich in the GO and KEGG analyses. We expanded the range of TRPM2-correlated genes (|*r*| > 0.3 and *p* < 0.05) and found that only the CC was enriched, indicating that TRPM2 may function mainly in the preribosome, lamellipodium, large subunit precursor of preribosome, and tertiary granule membrane (Supplementary Figure S5).

## Discussion

TRPM2, a highly Ca^2+^-permeable cation channel of the TRPM family, regulates cancer cell growth and survival. Although, in a small number of malignancies, the activation rather than the inhibition of TRPM2 has been reported to reduce tumor cell survival (Di et al., 2012), most of the studies considered that inhibition of the expression or function of TRPM2 resulted in decreased tumor proliferation and/or viability in several malignancies (Klumpp et al., 2016; Belrose and Jackson, 2018). In this study, for the first time, we analyzed the correlation between TRPM2 expression and its prognostic value in various cancers and the tumor immune cell infiltration in KIRC.

The expression of TRPM2 was elevated in various cancers compared to that in normal tissues (Figure 1). Survival analysis showed that a high TRPM2 expression was associated with poor prognosis in KIRC, LGG, OV, THYM, UVM, and LIHC patients (Figure 2) and better prognosis in BLCA, READ, UCEC, THCA, and STAD patients (Supplementary Figure S2). Among these cancer types, the expression level of TRPM2 was most relevant with the tumor subtype, stage, grade, and lymph node metastasis in KIRC (Figure 3). Moreover, TRPM2 was associated with TNM stage, especially with metastasis to lymph nodes and distant metastasis (Supplementary Table S1). These results suggested that TRPM2 plays a critical role in the progression and metastasis of KIRC, which was consistent with previous research studies showing that TRPM2 affects tumor growth and invasion and is correlated with poor prognosis in patients with breast, gastric, pancreatic, prostate, and head and neck cancers, melanoma, and neuroblastoma (Belrose and Jackson, 2018). Li et al. showed that TRPM2 mediated cancer cell migration through Ca^2+^ and Zn^2+^ (Li F. et al., 2016). In addition, TRPM2 downregulation inhibited the tumor growth of lung cancer in a xenograft NOD/SCID mouse model (Almasi et al., 2019).

Furthermore, the strong association of TRPM2 with the clinical characteristics of KIRC and the results of the multivariate analysis indicated that TRPM2 is an independent risk factor for OS in KIRC patients (Figures 3 and 4). According to research findings, when TRPM2-mediated calcium influx is inhibited, the mitochondria are dysfunctional, cellular bioenergetics is reduced, and the production of ROS is increased, thereby decreasing tumor growth and increasing chemotherapy sensitivity (Miller, 2019). Therefore, serum calcium was taken into account for evaluation. Intriguingly, we found that serum calcium was also an independent risk factor in KIRC patients (Figure 4). These data implicated aberrant intracellular Ca^2+^ signaling in the progression of KIRC.

Previously, TRPM2 was shown to regulate TME. It is expressed in monocytes and regulates CXCL2 production and neutrophil migration (Ambale-Venkatesh et al., 2017). Herein, we reported that the expression of TRPM2 was associated with several immune-infiltrating cells (B cells, CD8^+^ T cells, CD4^+^ T cells, macrophages, neutrophils, and dendritic cells) in KIRC through the correlation analysis in the TIMER database (Figure 5 and Table 1). Notably, an increased TRPM2 level was positively associated with Tregs and Th cells (Table 1). FOXP3 is a target for identifying Tregs in the TME, contributes to Treg differentiation, and mediates tumor immune escape (Sadlon et al., 2010). Consistently, our data revealed a positive association between the level of TRPM2 and FOXP3 (*r* = 0.516, after cell purity correction). Moreover, the positive correlation between TRPM2 and several Th cell (Th1, Th2, Tfh, and Th17) markers implied a significant regulatory role of TRPM2 in T-cell function in KIRC. These observations suggested that TRPM2 affects the prognosis of KIRC patients by regulating the recruitment of various T-cell subtypes.

Further investigation of the correlation of TRPM2 with T-cell inhibitory receptors indicated that TRPM2 was also positively correlated with T-cell exhaustion signature markers after cell purity correction (PDCD1: *r* = 0.583; CTLA4: *r* = 0.525; HAVCR2: *r* = 0.101; GZMB: *r* = 0.277; LAG3: *r* = 0.545) (Table 1). Collectively, these data indicated that TRPM2 might be the key factor that modulates T-cell exhaustion and inhibition of antitumor immune responses. This finding was consistent with that of a previous study showing that TRPM2 critically influences T-cell proliferation and pro-inflammatory cytokine secretion following polyclonal T-cell receptor stimulation (Melzer et al., 2012).

In order to investigate the molecular mechanism underlying TRPM2 regulating immune infiltration and tumor progression, we analyzed the network and focused on the specific functions and pathways of TRPM2-related genes in KIRC using GO and KEGG analyses. Based on TCGA-KIRC data, the TRPM2-associated genes with |*r*| > 0.5 and *p* < 0.05 were selected for enrichment. Interestingly, T-cell activation was ranked on top among biological processes (Figure 7). These results suggested that TRPM2 influences the progression of KIRC *via* T-cell activation.

In this study, we highlighted the role of TRPM2 as a potential prognostic marker by regulating immune cell infiltration in KIRC. Together with the results of the GO and KEGG enrichment analyses, the putative mechanism involved, but not limited to the regulation of T-cell activation and exhaustion, could provide clinical relevance of the function of TRPM2 in KIRC. However, the mechanism and clinical application of TRPM2 need to be substantiated further using *in vitro* and *in vivo* studies.

## Data Availability

The original contributions presented in the study are included in the article/Supplementary Material. Further inquiries can be directed to the corresponding author.

## Glossary

TRPM2: transient receptor potential melastatin-2
OS: overall survival
DFS: disease-free survival
RFS: relapse-free survival
TIME: tumor immune microenvironment
TPM: transcripts per kilobase million
KIRC: kidney renal clear cell carcinoma
ACC: adrenocortical carcinoma
BLCA: bladder urothelial carcinoma
BRCA: breast invasive carcinoma
CESC: cervical squamous cell carcinoma and endocervical adenocarcinoma
CHOL: cholangiocarcinoma
COAD: colon adenocarcinoma
DLBC: diffuse large B-cell lymphoma
ESCA: esophageal carcinoma
GBM: glioblastoma multiforme
HNSC: head and neck squamous cell carcinoma
KICH: kidney chromophobe
KIRP: kidney renal papillary cell carcinoma
LAML: acute myeloid leukemia
LGG: lower-grade glioma
LIHC: liver hepatocellular carcinoma
LUAD: lung adenocarcinoma
LUSC: lung squamous cell carcinoma
MESO: mesothelioma
OV: ovarian serous cystadenocarcinoma
PAAD: pancreatic adenocarcinoma
PCPG: pheochromocytoma and paraganglioma
PRAD: prostate adenocarcinoma
READ: rectum adenocarcinoma
SARC: sarcoma
SKCM: skin cutaneous melanoma
STAD: stomach adenocarcinoma
TGCT: testicular germ cell tumors
THCA: thyroid carcinoma
THYM: thymoma
UCEC: uterine corpus endometrial carcinoma
UCS: uterine carcinosarcoma
UVM: uveal melanoma
Th1: T helper cell 1
Th2: T helper cell 2
Treg: regulatory T cell
TAM: tumor-associated macrophage
NK cell: natural killer cell
Tfh: follicular helper T cells
PMN: polymorphonuclear neutrophils
HR: hazard ratio
CI: confidence interval
OR: odds ratio
GO: Gene Ontology
KEGG: Kyoto Encyclopedia of Genes and Genomes.

## References

[B1] Almasi S. Long C. Y. Sterea A. Clements D. R. Gujar S. El Hiani Y. (2019). TRPM2 Silencing Causes G2/M Arrest and Apoptosis in Lung Cancer Cells via Increasing Intracellular ROS and RNS Levels and Activating the JNK Pathway. Cell Physiol Biochem 52, 742–757. 10.33594/000000052 30933439

[B2] Ambale-Venkatesh B. Yang X. Wu C. O. Liu K. Hundley W. G. McClelland R. (2017). Cardiovascular Event Prediction by Machine Learning. Circ. Res. 121, 1092–1101. 10.1161/circresaha.117.311312 28794054 PMC5640485

[B3] Arora R. D. Limaiem F. (2021). “Renal Clear Cell Cancer,” in StatPearls [Internet]. Treasure Island, FL: StatPearls Publishing. Available at: https://www.ncbi.nlm.nih.gov/books/NBK563230/ (Updated September 28, 2021).

[B4] Belrose J. C. Jackson M. F. (2018). TRPM2: a Candidate Therapeutic Target for Treating Neurological Diseases. Acta Pharmacol. Sin 39, 722–732. 10.1038/aps.2018.31 29671419 PMC5943913

[B5] Binnewies M. Roberts E. W. Kersten K. Chan V. Fearon D. F. Merad M. (2018). Understanding the Tumor Immune Microenvironment (TIME) for Effective Therapy. Nat. Med. 24, 541–550. 10.1038/s41591-018-0014-x 29686425 PMC5998822

[B6] Chandrashekar D. S. Bashel B. Balasubramanya S. A. H. Creighton C. J. Ponce-Rodriguez I. Chakravarthi B. V. S. K. (2017). UALCAN: A Portal for Facilitating Tumor Subgroup Gene Expression and Survival Analyses. Neoplasia 19, 649–658. 10.1016/j.neo.2017.05.002 28732212 PMC5516091

[B7] Di A. Gao X.-P. Qian F. Kawamura T. Han J. Hecquet C. (2012). The Redox-Sensitive Cation Channel TRPM2 Modulates Phagocyte ROS Production and Inflammation. Nat. Immunol. 13, 29–34. 10.1038/ni.2171 PMC3242890 22101731

[B8] Díaz-Montero C. M. Rini B. I. Finke J. H. (2020). The Immunology of Renal Cell Carcinoma. Nat. Rev. Nephrol. 16, 721–735. 10.1038/s41581-020-0316-3 32733094

[B9] Gyorffy B. (2021). Survival Analysis across the Entire Transcriptome Identifies Biomarkers with the Highest Prognostic Power in Breast Cancer. Comput. Struct. Biotec 19, 4101–4109. 10.1016/j.csbj.2021.07.014 PMC8339292 34527184

[B10] Hopkins M. M. Feng X. Liu M. Parker L. P. Koh D. W. (2015). Inhibition of the Transient Receptor Potential Melastatin-2 Channel Causes Increased DNA Damage and Decreased Proliferation in Breast Adenocarcinoma Cells. Int. J. Oncol. 46, 2267–2276. 10.3892/ijo.2015.2919 25760245 PMC4383028

[B11] Klumpp D. Misovic M. Szteyn K. Shumilina E. Rudner J. Huber S. M. (2016). Targeting TRPM2 Channels Impairs Radiation-Induced Cell Cycle Arrest and Fosters Cell Death of T Cell Leukemia Cells in a Bcl-2-dependent Manner. Oxid Med. Cell Longev 2016, 8026702. 10.1155/2016/8026702 26839633 PMC4709732

[B12] Lange I. Yamamoto S. Partida-Sanchez S. Mori Y. Fleig A. Penner R. (2009). TRPM2 Functions as a Lysosomal Ca2+-Release Channel in Beta Cells. Sci. Signal. 2, ra23. 10.1126/scisignal.2000278 19454650 PMC2779714

[B13] Li B. Severson E. Pignon J. C. Zhao H. Li T. Novak J. (2016a). Comprehensive Analyses of Tumor Immunity: Implications for Cancer Immunotherapy. Genome Biol. 17, 174. 10.1186/s13059-016-1028-7 27549193 PMC4993001

[B14] Li F. Abuarab N. Sivaprasadarao A. (2016b). Reciprocal Regulation of Actin Cytoskeleton Remodelling and Cell Migration by Ca2+ and Zn2+: Role of TRPM2 Channels. J. Cell Sci 129, 2016–2029. 10.1242/jcs.179796 27068538

[B15] Li T. Fan J. Wang B. Traugh N. Chen Q. Liu J. S. (2017). TIMER: A Web Server for Comprehensive Analysis of Tumor-Infiltrating Immune Cells. Cancer Res. 77, E108–E110. 10.1158/0008-5472.CAN-17-0307 29092952 PMC6042652

[B16] Li T. Fu J. Zeng Z. Cohen D. Li J. Chen Q. (2020). TIMER2.0 for Analysis of Tumor-Infiltrating Immune Cells. Nucleic Acids Res. 48, W509–W514. 10.1093/nar/gkaa407 32442275 PMC7319575

[B17] Linehan W. M. (2012). Genetic Basis of Kidney Cancer: Role of Genomics for the Development of Disease-Based Therapeutics. Genome Res. 22, 2089–2100. 10.1101/gr.131110.111 23038766 PMC3483538

[B18] Marquardt A. Solimando A. G. Kerscher A. Bittrich M. Kalogirou C. Kübler H. (2021). Subgroup-Independent Mapping of Renal Cell Carcinoma-Machine Learning Reveals Prognostic Mitochondrial Gene Signature beyond Histopathologic Boundaries. Front. Oncol. 11, 621278. 10.3389/fonc.2021.621278 33791209 PMC8005734

[B19] Melzer N. Hicking G. Göbel K. Wiendl H. (2012). TRPM2 Cation Channels Modulate T Cell Effector Functions and Contribute to Autoimmune CNS Inflammation. Plos One 7, e47617. 10.1371/journal.pone.0047617 23077651 PMC3470594

[B20] Miller B. A. (2019). TRPM2 in Cancer. Cell Calcium 80, 8–17. 10.1016/j.ceca.2019.03.002 30925291 PMC6545160

[B21] Mittal M. Nepal S. Tsukasaki Y. Hecquet C. M. Soni D. Rehman J. (2017). Neutrophil Activation of Endothelial Cell-Expressed TRPM2 Mediates Transendothelial Neutrophil Migration and Vascular Injury. Circ. Res. 121, 1081–1091. 10.1161/circresaha.117.311747 28790198 PMC5640489

[B22] Paricio-Montesinos R. Schwaller F. Udhayachandran A. Rau F. Walcher J. Evangelista R. (2020). The Sensory Coding of Warm Perception. Neuron 106, 830–841. 10.1016/j.neuron.2020.02.035 32208171 PMC7272120

[B23] Park Y. R. Chun J. N. So I. Kim H. J. Baek S. Jeon J. H. (2016). Data-driven Analysis of TRP Channels in Cancer: Linking Variation in Gene Expression to Clinical Significance. Cancer Genomics Proteomics 13, 83–90. 26708603

[B24] Ru B. Wong C. N. Tong Y. Zhong J. Y. Zhong S. S. W. Wu W. C. (2019). TISIDB: an Integrated Repository portal for Tumor-Immune System Interactions. Bioinformatics 35, 4200–4202. 10.1093/bioinformatics/btz210 30903160

[B25] Sadlon T. J. Wilkinson B. G. Pederson S. Brown C. Y. Bresatz S. Gargett T. (2010). Genome-Wide Identification of Human FOXP3 Target Genes in Natural Regulatory T Cells. J.I. 185, 1071–1081. 10.4049/jimmunol.1000082 20554955

[B26] Sumoza-Toledo A. Lange I. Cortado H. Bhagat H. Mori Y. Fleig A. (2011a). Dendritic Cell Maturation and Chemotaxis Is Regulated by TRPM2-Mediated Lysosomal Ca2+ Release. Faseb J. 25, 3529–3542. 10.1096/fj.10-178483 21753080 PMC3177582

[B27] Sumoza-Toledo A. Penner R. (2011b). TRPM2: a Multifunctional Ion Channel for Calcium Signalling. J. Physiol-london 589, 1515–1525. 10.1113/jphysiol.2010.201855 21135052 PMC3099011

[B28] Sun L. Yau H. Y. Wong W. Y. Li R. A. Huang Y. Yao X. (2012). Role of TRPM2 in H(2)O(2)-induced Cell Apoptosis in Endothelial Cells. Plos One 7, e43186. 10.1371/journal.pone.0043186 22916222 PMC3423428

[B29] Tan C.-H. McNaughton P. A. (2016). The TRPM2 Ion Channel Is Required for Sensitivity to Warmth. Nature 536, 460–463. 10.1038/nature19074 27533035 PMC5720344

[B30] Tang Z. Li C. Kang B. Gao G. Li C. Zhang Z. (2017). GEPIA: a Web Server for Cancer and normal Gene Expression Profiling and Interactive Analyses. Nucleic Acids Res. 45, W98–W102. 10.1093/nar/gkx247 28407145 PMC5570223

[B31] Vuong L. Kotecha R. R. Voss M. H. Hakimi A. A. (2019). Tumor Microenvironment Dynamics in Clear-Cell Renal Cell Carcinoma. Cancer Discov. 9, 1349–1357. 10.1158/2159-8290.cd-19-0499 31527133 PMC6774890

[B32] Wang Y. Zhang Y. Wang P. Fu X. Lin W. (2020). Circular RNAs in Renal Cell Carcinoma: Implications for Tumorigenesis, Diagnosis, and Therapy. Mol. Cancer 19, 149. 10.1186/s12943-020-01266-7 33054773 PMC7559063

[B33] Zeng Q. Zhang W. Li X. Lai J. Li Z. (2020). Bioinformatic Identification of Renal Cell Carcinoma Microenvironment-Associated Biomarkers with Therapeutic and Prognostic Value. Life Sci. 243, 117273. 10.1016/j.lfs.2020.117273 31926244

