# Supplementary material for: Identification of TRPM2 as a Marker Associated With Prognosis and Immune Infiltration in Kidney Renal Clear Cell Carcinoma
Source: Front Mol Biosci. 2022 Jan 5;8:774905. doi: 10.3389/fmolb.2021.774905 (PMC8769242; doi:10.3389/fmolb.2021.774905)
Supplement: Supplementary file 1 [file DataSheet2.docx]

Supplementary Figures


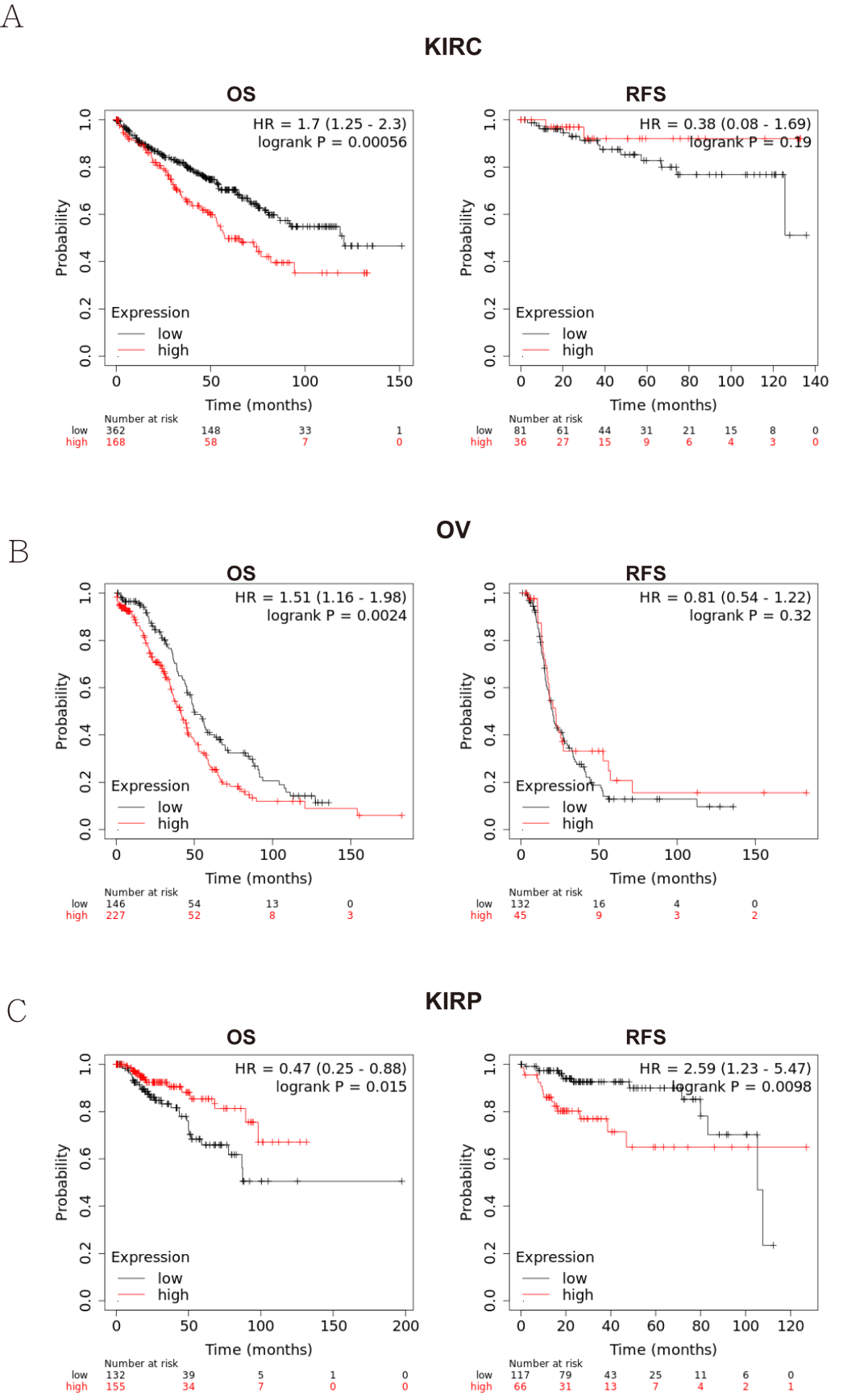


Supplementary Figure 1

Kaplan-Meier survival curves comparing the high and low expression of TRPM2 in KIRC (A), OV (B) and KIRP (C) patients. Red curve represented patients with higher expression of TRPM2. Black curve represented patients with lower expression of TRPM2.


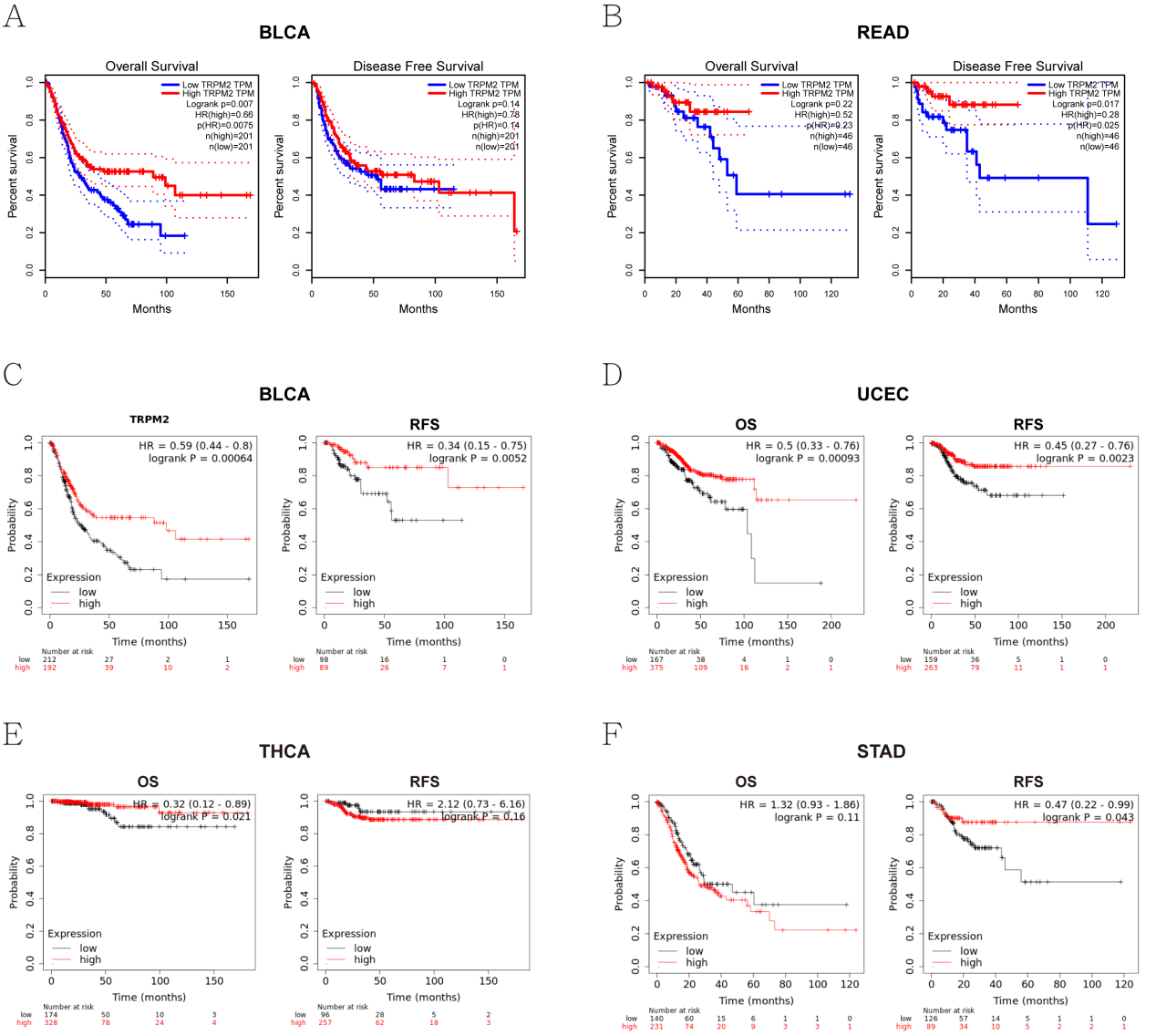


Supplementary Figure 2

The association of TRPM2 expression and prognosis of BLCA (A, C), READ (B), UCEC (D), THCA (E) and STAD (F) patients using GEPIA (A, B) and Kaplan Meier-Plotter (C-F) database, respectively.


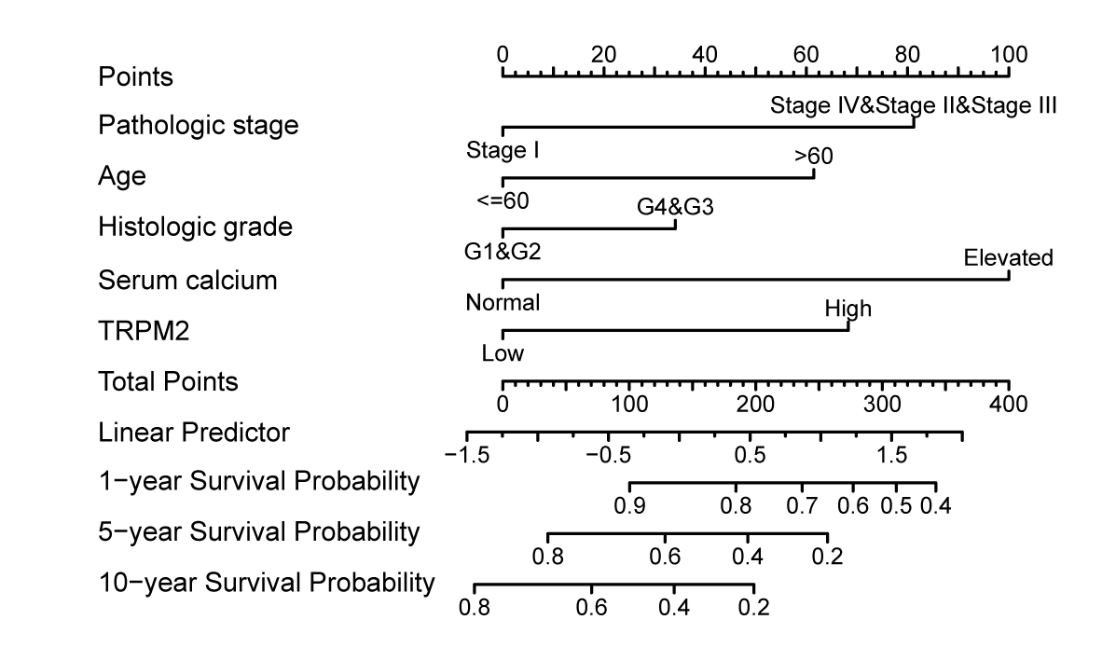


Supplementary Figure 3

Nomogram showing the pathologic stages, age, histologic grade, serum calcium, TRPM2 expression and 1year, 5year, and 10 year OS of KIRC patients using the Cox regression algorithm.


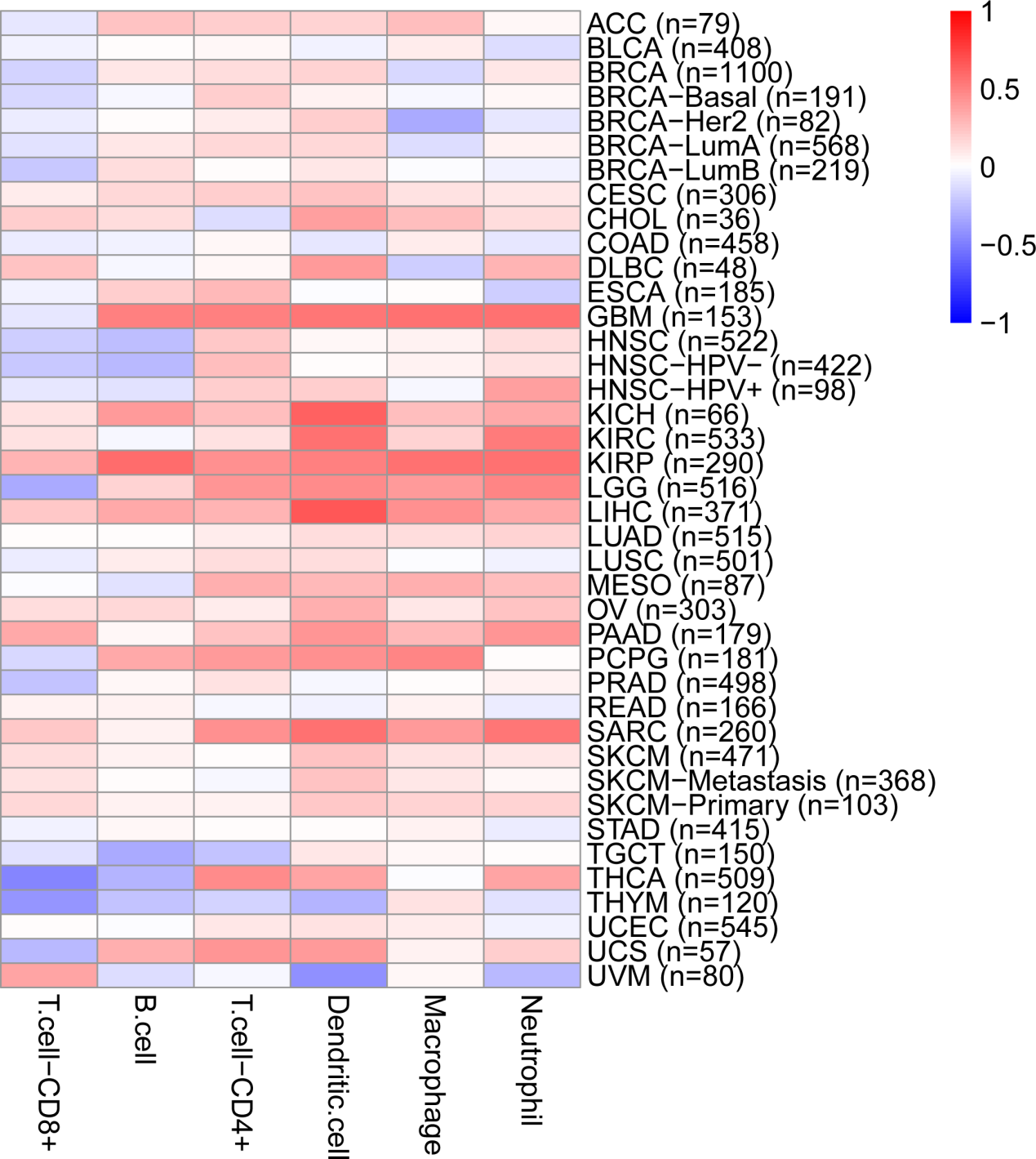


Supplementary Figure 4

Heatmap showing the correlation of TRPM2 level with the infiltration level of B cells, CD8^+^ T cells, CD4^+^ T cells, macrophages, neutrophils and dendritic cells across human cancers using TIMER 2.0 database.


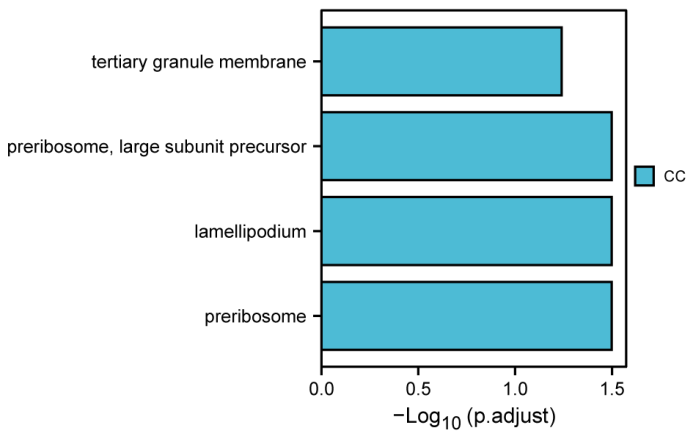


Supplementary Figure 5

GO and KEGG enrichment analysis of TRPM2-related genes in BLCA.

TRPM2-related genes (｜r｜>0.3 and *p*<0.05) in BLCA were subjected to GO classification and KEGG pathway enrichment analysis. Pearson’s correlation coefficient between TRPM2 and each of the genes of interest was calculated.


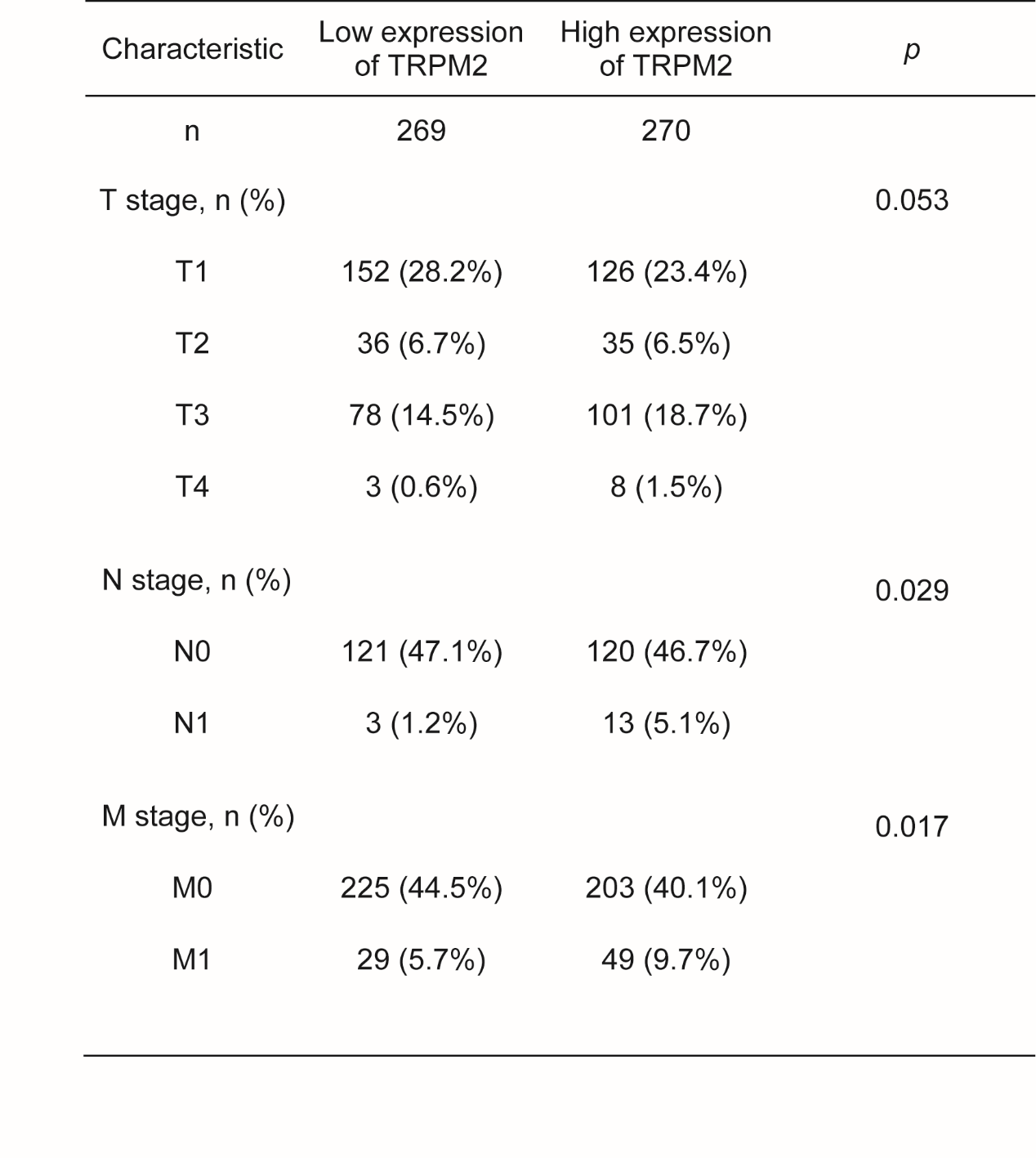


Supplemental Table 1

Correlation of TRPM2 mRNA level with clinical characteristic parameter (T stage, N stage and M stage) of KIRC using TCGA database. Chisq.test was used to determine *p* value.
